# Spatial Scaling of the Profile of Selective Attention in the Visual Field

**DOI:** 10.1371/journal.pone.0162190

**Published:** 2016-09-08

**Authors:** Matthew A. Gannon, Ashley A. Knapp, Thomas G. Adams, Stephanie M. Long, Nathan A. Parks

**Affiliations:** 1 Department of Psychological Science, University of Arkansas, Fayetteville, Arkansas, United States of America; 2 Department of Psychiatry, Yale University, New Haven, Connecticut, United States of America; University of Groningen, NETHERLANDS

## Abstract

Neural mechanisms of selective attention must be capable of adapting to variation in the absolute size of an attended stimulus in the ever-changing visual environment. To date, little is known regarding how attentional selection interacts with fluctuations in the spatial expanse of an attended object. Here, we use event-related potentials (ERPs) to investigate the scaling of attentional enhancement and suppression across the visual field. We measured ERPs while participants performed a task at fixation that varied in its attentional demands (attentional load) and visual angle (1.0° or 2.5°). Observers were presented with a stream of task-relevant stimuli while foveal, parafoveal, and peripheral visual locations were probed by irrelevant distractor stimuli. We found two important effects in the N1 component of visual ERPs. First, N1 modulations to task-relevant stimuli indexed attentional selection of stimuli during the load task and further correlated with task performance. Second, with increased task size, attentional modulation of the N1 to distractor stimuli showed a differential pattern that was consistent with a scaling of attentional selection. Together, these results demonstrate that the size of an attended stimulus scales the profile of attentional selection across the visual field and provides insights into the attentional mechanisms associated with such spatial scaling.

## Introduction

Visual selective attention is the neural process that increases the signal-to-noise ratio (SNR) of behaviorally relevant visual representations via the enhancement of task-relevant stimuli and suppression of task-irrelevant (distractor) stimuli. Extensive literature has examined the neural mechanisms of visual selective attention and the behavioral and perceptual consequences associated with engaging those mechanisms [1]-[5]. Despite the expansiveness of this literature, there remains a paucity of knowledge regarding the spatial dynamics of visual selective attention during the sustained performance of a visual task at fixation; studies of visual selective attention draw largely upon stimuli of fixed size presented in peripheral retinal positions. However, interacting with objects in natural environments rarely involves stimuli of a constant visual angle anchored invariably to a single depth plane. Rather, the absolute visual angle of an attended object is in constant flux as an observer moves closer or further from the object, or the object moves toward or away from the observer. Furthermore, visual behavior in natural environments typically involves foveating an attended object amongst a cluttered scene of numerous visual stimuli, and maintaining fixation on that object while it remains behaviorally relevant. Though a number of models of attention have emphasized the importance of spatial scaling in processes of visual selection [6]-[10], these studies have focused on peripheral visual attention and have not specifically investigated the spatial scaling of selection at fixation. To date, the operation of neural mechanisms of attentional selection under naturalistic viewing conditions has not been thoroughly investigated. Here, we provide a step toward understanding the processes of attentional selection under pseudo-naturalistic viewing by examining how fixated stimuli of varying sizes impact the spatial scaling of attentional selection.

An effective and well-established laboratory paradigm for examining visual selective attention at fixation involves manipulation of the attentional load of a centrally presented task-relevant stimulus (also perceptual load) [5], [11–14]. Attentional load refers to increasing the attentional demands of a task through stimulus parameters, typically through a conjunction of stimulus dimensions [11], [15]. Previous work has established that increasing the attentional load of a central visual task leads to enhancement of task-relevant representations, while simultaneously suppressing irrelevant peripheral visual representations [16]-[22]. To investigate the effects of the spatial extent of an attended stimulus on the scaling of attentional enhancement and suppression, we varied the attentional load (low versus high load) and task-relevant object size (attentional field size; 1.0° or 2.5°) in this central load task. We examined early visual components of the ERP (P1 and N1) for evidence of the scaling of attentional enhancement and suppression, as these components are well known to modulate with attention [23], [24] and originate within extrastriate visual networks [25]. Using P1 and N1 amplitude as indices of attentional selection, we sought to determine whether the spatial pattern of attentional enhancement and suppression across the visual field scales according to the size of a task-relevant stimulus at fixation.

## Materials and Methods

### Subjects

Thirty-eight subjects (twenty females; mean age = 20 years, *SD* = 2.6 years) were recruited from the University of Arkansas undergraduate subject pool. Subjects were naïve to the purposes of the study and self-reported having normal or corrected-to-normal vision. Out of this sample, three subjects were subsequently excluded for poor behavioral performance (performance less than 2 SD below sample mean), five were eliminated for excessive ocular artifact (> 20% of trials), and six subjects were excluded for poor signal-to-noise (SNR) of probe ERPs according to the method described by Parks, Gannon, Young, and Long [26]. Subject exclusions based on SNR were determined using the lower bound of SNR confidence intervals (*SNR*_*LB*_) estimated from a bootstrap resampling procedure (50,000 bootstraps) and using an inclusion threshold of 3.0 dB [26]. Subjects received research credits toward their Psychology course requirements as compensation for their participation. All procedures were approved by the University of Arkansas Institutional Review Board and each subject gave written informed consent prior to their participation.

### Stimuli and Procedure

Experimentation was conducted in a laboratory testing room under low levels of ambient illumination. Stimuli were presented on a 21-inch CRT monitor (85 Hz vertical refresh, 1024 × 768 resolution), electrically shielded in a grounded aluminum Faraday cage. A viewing distance of 57 cm was maintained with a chinrest. The duration of the experiment was approximately three hours.

**Fig 1:**
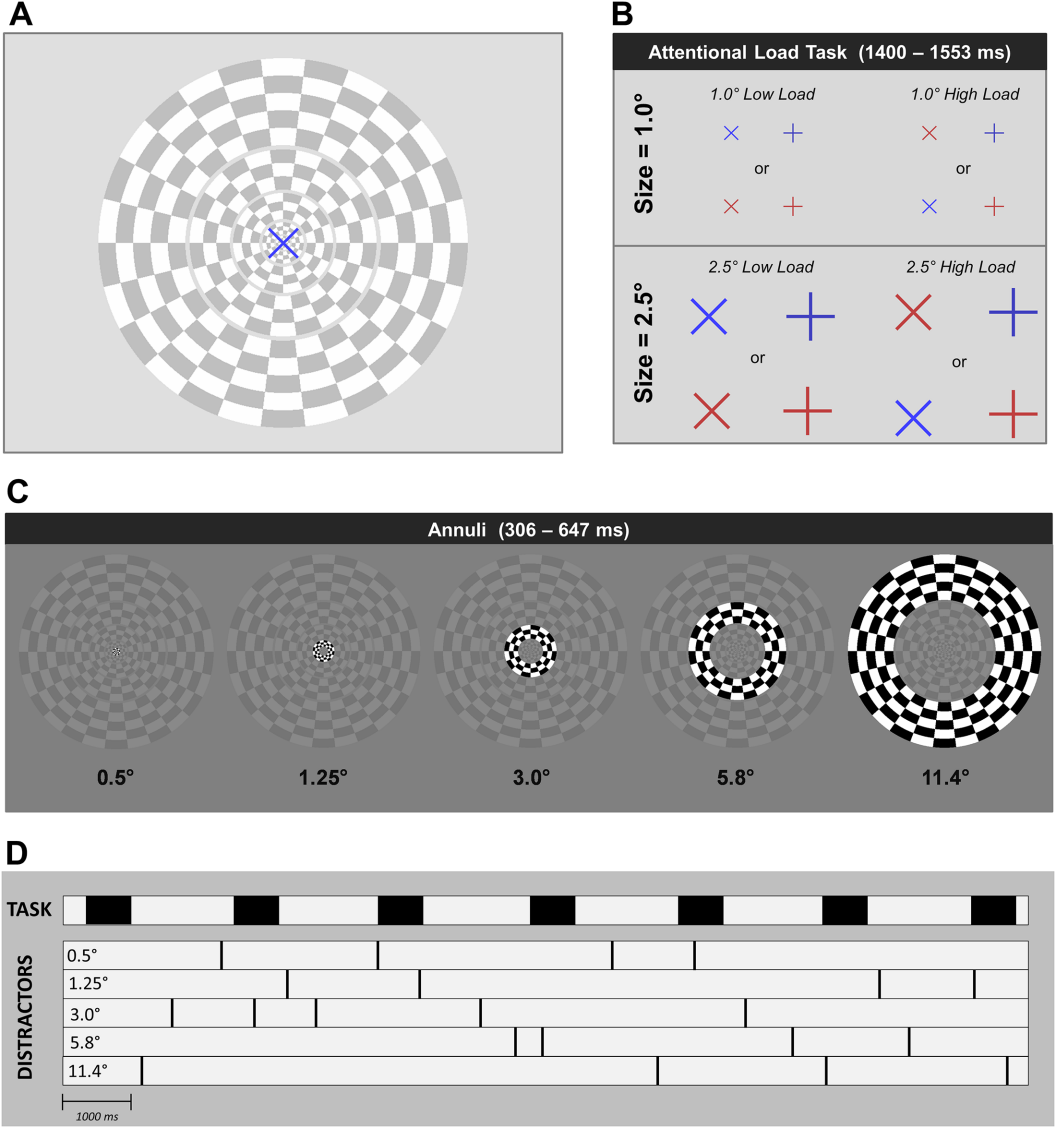
(A) Stimuli of the attentional load task were superimposed upon a set of five irrelevant checkerboard annuli distractors, which independently contrast reversed. (B) Attentional load task consisted of a singleton identification or conjunction of two features. (C) During performance of this task, one of five irrelevant distractor checkerboard annuli was randomly selected for contrast reversal. (D) Illustration and example of task and distractor onset timing over a hypothetical fourteen second period during a given experimental block. Filled in black areas in the area denoted ‘TASK’ represent onsets and durations of task-relevant stimuli. Black tick marks in the area denoted ‘DISTRACTORS’ represent the occurrence of a contrast reversal at the five distractor eccentricities.

Subjects performed a go/no-go task under low or high attentional load while irrelevant foveal, parafoveal, and peripheral distractor probes contrast reversed (Fig 1A). Subjects maintained fixation on a central yellow fixation cross (0.2° diameter) while task stimuli onset every 1400–1553 ms and persisted for a duration of 647 ms. Task stimuli were “×” and “+” shapes, either blue or red in color. Thus, there were four stimulus types of the attentional load task: blue “×”, red “×”, blue “+”, and red “+”. Subjects performed a go/no-go task according to the assignment of two of these four stimuli as targets. Subjects were instructed to press a button when either of the two assigned targets onset but withhold responses to the two non-target stimuli. There was an equal proportion of targets and non-target stimuli (50/50 go/no-go task). Attentional load was manipulated between blocks of trials by the assignment of the two target stimuli at the beginning of the block (Fig 1B). In low attentional load blocks targets were assigned as stimuli of identical color (e.g., blue “×” and blue “+”), whereas targets in high load blocks were assigned as a conjunction between stimulus color and orientation (e.g., blue “×” and red “+”). Discrimination of targets based on feature singletons versus feature conjunctions is a well-established method for manipulating attentional demands while holding stimulus properties constant [11], [15]. That is, this approach to manipulating attention uses identical stimuli and varies only the attentional demands of the task being performed on these stimuli. The size of the task-relevant stimulus was also manipulated parametrically with attentional load by setting its visual angle to a diameter of either 1.0° or 2.5° (Fig 1B). Thus, the combinations of attentional load and task size resulted in four blocked task conditions: *1*.*0° low load*, *1*.*0° high load*, *2*.*5° low load*, and *2*.*5° high load*. The inclusion of low load and high load manipulations within the two manipulations of task size ensured that attentional effects could be compared across conditions despite the low-level stimulus differences (e.g., task size) between these conditions.

Stimuli of the previously described attentional load tasks were superimposed upon an array of five task-irrelevant checkerboard annuli positioned at five eccentricities in the visual field. These checkerboard annuli were scaled for cortical magnification according to the method described by Carrasco and Frieder [27] and were positioned such that their outer radii annuli fell at eccentricities of 0.5°, 1.25°, 3.0°, 5.8°, and 11.4° (Fig 1C). The inner and outer borders of abutting distractor annuli were spaced by 0.2°. Crucially, 1.0° task-relevant stimuli overlapped in visual space with only the 0.5° annulus whereas 2.5° load stimuli overlapped in visual space with both 0.5° and 1.25° annuli. Though small stimulus differences in 0.5° and 1.25° distractor annuli were introduced by the task size manipulation, the inclusion of low load and high load conditions within each task size manipulations allowed low-level stimulus differences to be controlled. During experimental blocks one of the five distractor annuli underwent a single contrast reversal every 306 to 647 ms. Over the course of an experimental block each of the five annuli underwent 43 contrast reversals (215 total reversals per block). The order of these 215 contrast reversals was randomly shuffled for each experimental block.

Prior to each block, subjects were shown an image of the assigned “go” targets for that block and pressed a button when they were ready to begin the block. A screen containing the five checkerboard annuli and central fixation point then onset. Following a 1400 ms fixation period, a task-relevant stimulus onset (647 ms duration), with a new stimulus appearing every 1400 to 1553 ms. Following four such task stimuli, distractor annuli began to contrast reverse with one of the five rings undergoing a single reversal every 306 to 647 ms. An illustration of task and distractor stimulus timing is provided in Fig 1D. Task and distractor stimuli continued to onset in this fashion through 124 presentations of go/no-go stimuli and 215 contrast reversals of distractor checkerboard annuli (43 reversals per annulus). Participants completed 16 experimental blocks, equally divided between task conditions of *1*.*0° low load*, *1*.*0° high load*, *2*.*5° low load*, and *2*.*5° high load*. The order of task stimuli and distractor stimuli was randomly shuffled for each block. The order of blocks was randomly shuffled for each participant and consisted of eight different combinations of potential targets (2 load conditions × 2 colors × 2 target sizes), each presented twice during the experiment.

### Electrophysiological Recording and Analysis

Continuous EEG was recorded with a 64-channel BrainAmp DC amplifier equipped with the ActiCap active electrode system (Brain Products, Munich, Germany). Sixty-one scalp electrodes were placed in accordance with the 10–10 system at positions AF3/4, AF7/8, Fz, F1/2, F3/4, F5/6, F7/8, FCz, FC1/2, FC3/4, FC5/6, FT7/8, Cz, C1/2, C3/4, C5/6, CPz, CP1/2, CP3/4, CP5/6, T7/8, TP7/8, Pz, P1/2, P3/4, P5/6, P7/8, POz, PO3/4, PO7/8, PO9/10, Oz, O1/2, and M1/2. Electrooculogram (EOG) was acquired from four additional electrodes positioned on the left and right canthi and above and below the left eye. EEG and EOG data were recorded online in reference to electrode FCz, low-pass filtered at 250 Hz, and digitally sampled at 1000 Hz. Offline, scalp-recorded EEG was re-referenced to the average of the left and right mastoid channels. Two bipolar EOG channels were calculated to form horizontal (HEOG) and vertical (VEOG) EOG as the difference between channels on the left and right canthi and channels above and below the left eye channels, respectively. Continuous EEG and EOG data was digitally band-pass filtered from 0.1 to 30 Hz (24 dB/octave) and corrected for VEOG and HEOG artifacts using a standard regression procedure [28]. Continuous EEG was segmented into 800 ms epochs, -200 to 600 ms relative to the onset of attentional load task stimuli or checkerboard annulus contrast reversal. Epochs were baseline corrected according to the 200 ms pre-stimulus interval and rejected as an artifact if voltage in any scalp recorded channel exceeded ±150 μV.

**Fig 2:**
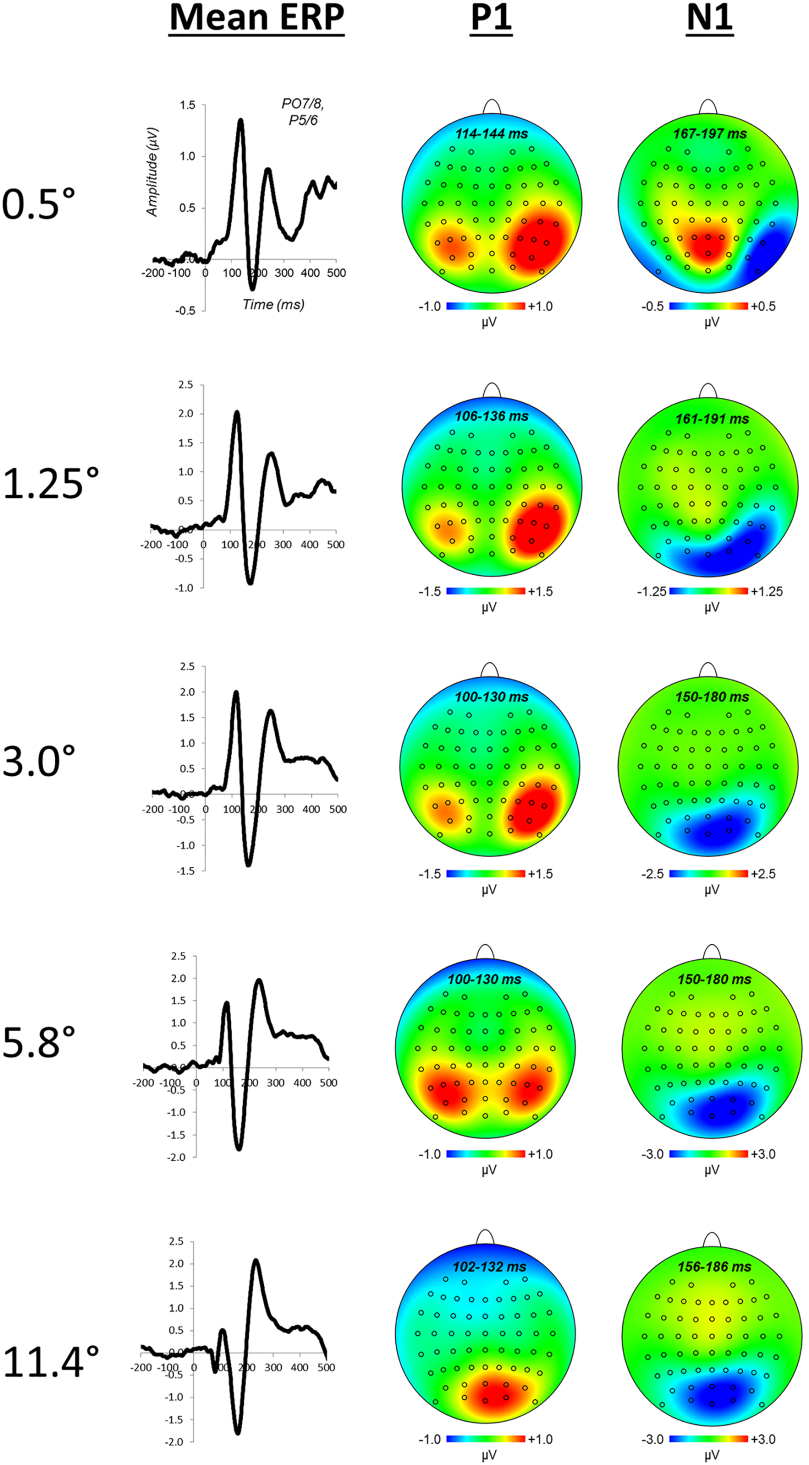
Mean ERPs for each of the five annulus distractor eccentricities and associated scalp distributions for P1 and N1 components. Waveforms are pool across electrodes P5/6 and PO7/8.

P1 and N1 components of visual evoked potentials were quantified as the average voltage within a specified time window. Time windows for P1 and N1 were determined individually for each class of stimulus (i.e., task stimulus and 0.5°, 1.25°, 3.0°, 5.8°, and 11.4° annuli) from grand average ERPs collapsed across all experimental manipulations. P1 and N1 peaks were quantified by centering a 31 ms time window on the peak latency of grand average waveforms (15 ms width on either side of the peak). Peak latencies for P1 and N1 components of attentional load task ERPs were 142 and 204 ms, respectively. For distractor annuli ERPs, the P1 peaked at 129 ms (0.5°), 121 ms (1.25°), 115 ms (3.0°), 115 ms (5.8°), and 117 ms (11.4°). The N1 peaked at 182 ms (0.5°), 176 ms (1.25°), 165 ms (3.0°), 165 ms (5.8°), and 171 ms (11.4°). The scalp distribution of the grand average waveforms at each eccentricity was used to select electrodes for statistical analyses (Fig 2). For eccentricities of 0.5° and 1.25° electrodes P5 and PO7 were pooled in the left hemisphere and P6 and PO8 for right hemisphere. The more eccentric distractors at 3.0°, 5.8°, and 11.4° exhibited greater medial distributions in scalp topographies (Fig 2). As such, a more medial set of electrodes were pooled for these eccentricities: O1 and PO3 for the left hemisphere and O2 and PO4 for the right hemisphere.

Behavioral performance (response time and *d’*) in the attentional load task was analyzed with 2 × 2 repeated measures ANOVAs with factors of attentional field size (size: 1.0° or 2.5°) and attentional load (load: low or high). P1 and N1 components in the attentional load task were analyzed with separate 2 × 2 × 2 repeated measures analysis of variance (ANOVA) with factors of hemisphere (left or right), attentional field size (size: 1.0° or 2.5°), and attentional load (load: low or high). P1 and N1 components for task-irrelevant distractor annuli were submitted to a 2 × 2 × 2 × 5 repeated measures ANOVAs with factors of hemisphere (left or right), task size (size: 1.0° or 2.5°), attentional load (load: low or high), and annulus eccentricity (eccentricity: 0.5°, 1.25°, 3.0°, 5.8°, and 11.4°). When appropriate, degrees of freedom were Huynh-Feldt corrected for violations of sphericity. Planned polynomial interaction contrasts were used to examine trends in annuli ERPs for the impact of task size on the pattern of attentional load modulations across the visual field. These contrasts tested for linear, quadratic, cubic, and quartic trends in the size × load × eccentricity interaction for P1 and N1 components.

## Results

**Fig 3:**
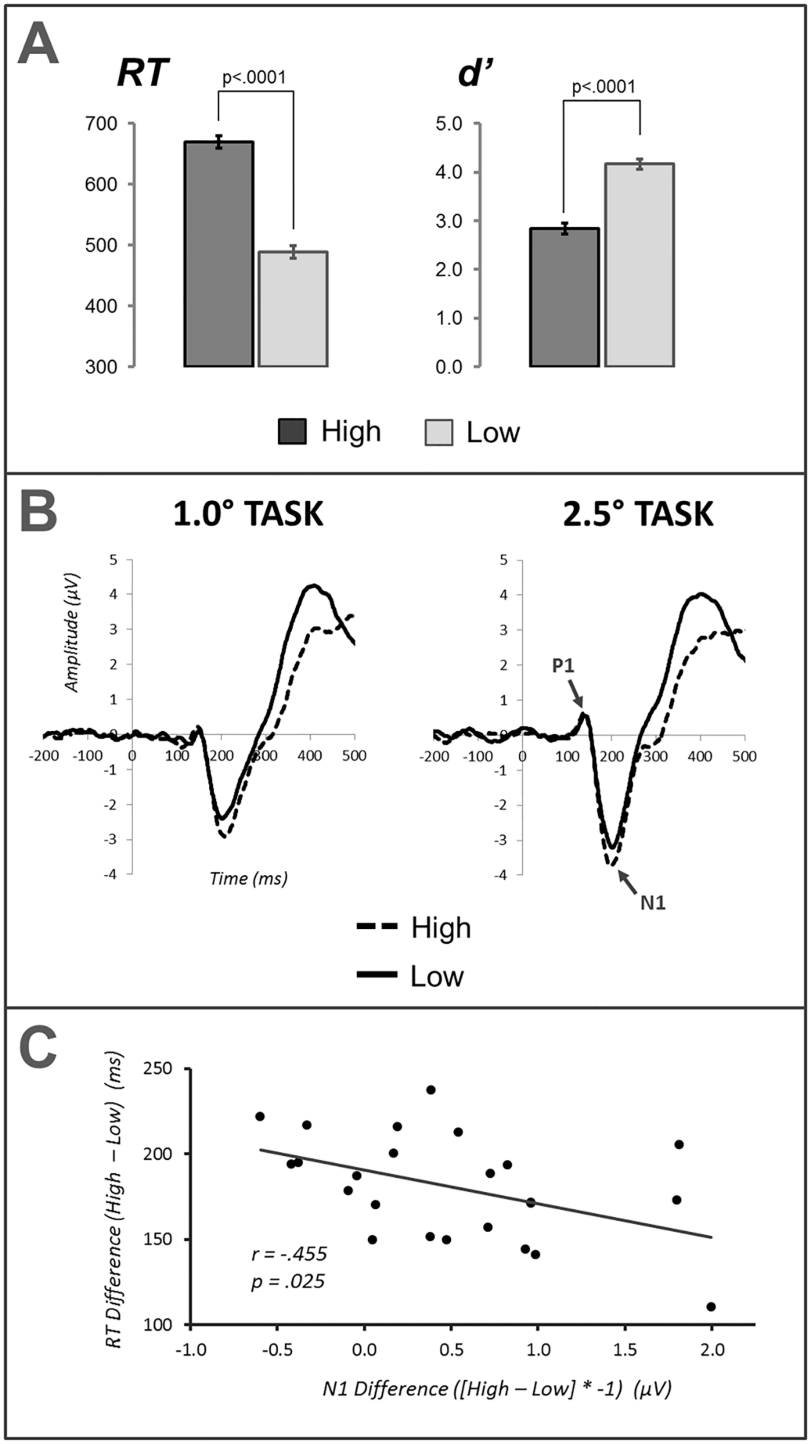
(A) Response time and *d’* for high and low load conditions in the attentional load task, (B) ERPs to high and low load task stimuli pooled across electrode P5/6 and PO7/8, and (C) scatterplot and correlation of N1 difference amplitude and RT difference scores in the attentional load task. Note that N1 difference scores in this correlation have been multiplied by -1 for convenience of visualization and interpretation. Error bars represent one SEM.

Behavioral results were consistent with standard findings of attentional load [11], [14]. Response times (RTs) were significantly slower under high load than low load, *F*_(1,23)_ = 832.6, *p* < .0001, and *d’* was significantly lower under high load relative to low load, *F*_(1,23)_ = 111.2, *p* < .0001 (Fig 3A). There were no significant effects of task size on behavioral performance.

Omnibus ANOVAs for P1 and N1 components for attentional load task ERPs revealed no effects of attentional load on P1 amplitudes but a significant main effect of load in the N1 component, *F*_(1,23)_ = 12.29, *p* < .005. This N1 effect resulted from greater amplitude under high load, indicating a potentiated visual extrastriate response within task-relevant stimulus representations (Fig 3B). To examine the relationship between behavioral performance and N1 amplification, we correlated RT and *d’* difference scores with N1 difference amplitudes (high load–low load). For convenience of visualization and interpretation, N1 difference amplitudes used in this correlation (and all subsequent analyses) were multiplied by -1, such that N1 enhancement yielded positive values and suppression yielded negative values. The correlation between RT and N1 difference scores revealed a significant negative correlation, *r*_(22)_ = -.455, *p* = .025 (Fig 3C); greater N1 potentiation was associated with faster RTs. This relationship is consistent with improved signal-to-noise in task-relevant visual representations, or more efficacious selection of these representations, leading to an associated improvement in performance.

**Fig 4:**
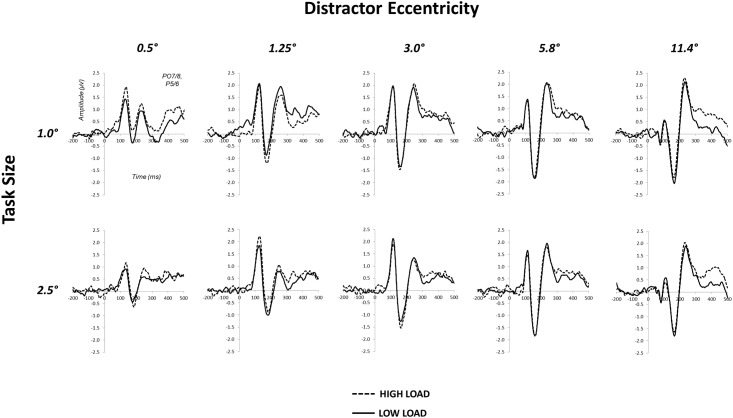
Mean ERPs at each distractor eccentricity for the two task sizes under low and high load conditions. Waveforms are pool across electrodes P5/6 and PO7/8.

**Fig 5:**
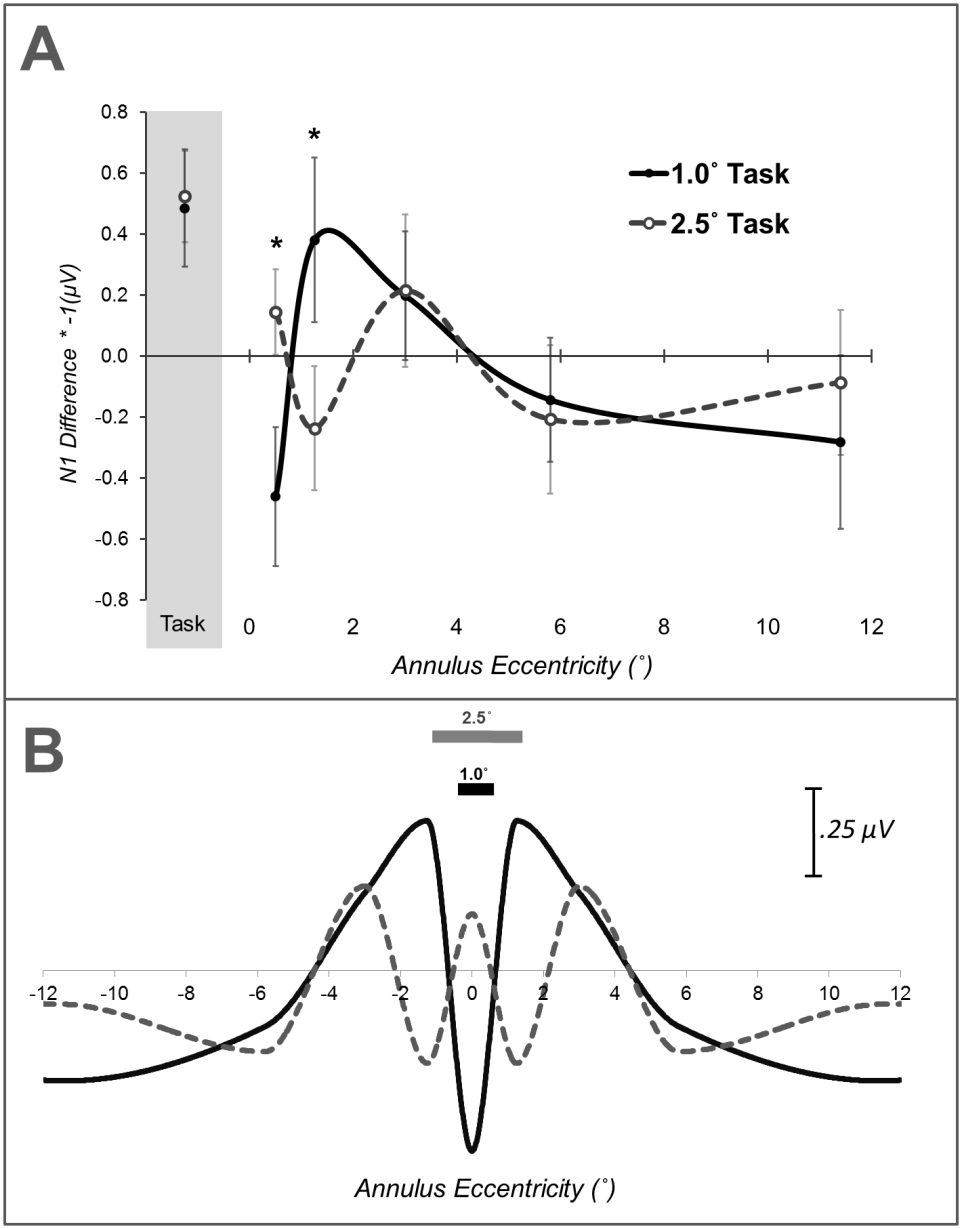
(A) N1 difference amplitudes for irrelevant checkerboard annuli in 1.0° and 2.5° load tasks, plotted as a function of probe position in the visual field. For convenience of visualization, N1 difference amplitudes are multiplied by -1 such that positive values reflect enhancement and negative values reflect suppression. (B) Interpolated representation of a one-dimensional cross-section of the spatial profiles of N1 modulation of irrelevant distractors during performance of 1.0° and 2.5° attentional load task. One-dimensional profiles were generated by cubic interpolation of N1 modulation data mirrored around the 0.5° annulus.

Plots of mean distractor waveforms and attentional load differences between distractor waveforms are shown in Figs 2 and 4, respectively. The omnibus ANOVA for P1 did not reveal any effects of attentional load. The omnibus ANOVA for N1 amplitudes yielded a significant hemisphere × load × eccentricity interaction, *F*_(4,92)_ = 2.55, *p* = .044. Follow-up contrasts comparing load differences between task size at each annulus eccentricity were run to further examine the nature of this size × load × eccentricity interaction. These contrasts revealed significant differences at distractor eccentricities of 0.5° and 1.25°, *t*_(23)_ = 2.51, *p* = .020 and *t*_(23)_ = -2.19, *p* = .039, respectively. Planned polynomial contrasts for the N1 amplitude size × load × eccentricity interaction further evidenced a differential geometric pattern of size-dependent effects of attentional load through a significant quadratic trend, *F*_(1,23)_ = 5.02, *p* = .035. The presence of a size × load × eccentricity interaction, outcomes of follow-up t-tests, and a significant quadratic trend clearly demonstrate a differential geometric pattern of attentional load across the five visual field distractors due to the size of the attended stimulus. The nature of these differential patterns is apparent by the inspection of N1 difference amplitudes across irrelevant distractor eccentricities (Fig 5A), and visualizations of interpolated 1-dimensional spatial cross-sections (Fig 5B). Load-dependent N1 suppression was apparent within the spatial boundaries of the task-relevant stimulus, for ring annuli most proximal to the edge of the attended stimulus (0.5° annulus for the 1.0° task and 1.25° annulus for the 2.5° task). N1 enhancement was indicated for stimuli at the most proximal position outside the spatial boundaries of the attended stimulus (1.25° annulus for the 1.0° task and 3.0° annulus for the 2.5° task).

## Discussion

A complete and ecologically valid account of visual selective attention must be capable of integrating proposed mechanisms of attentional selection with natural visual behaviors. Here, we sought to better understand the spatial scaling of selective attention necessary for maintaining selection of a fixated object varying in absolute visual angle and presented amongst a crowded scene of irrelevant distractor stimuli. We measured visual cortical responses (ERPs) evoked by both task-relevant stimuli and task-irrelevant distractor probes during performance of a sustained attention task at fixation. To investigate the spatial scaling of visual selective attention, we manipulated the size of task-relevant stimuli (1.0° or 2.5° in diameter) and measured alterations in the pattern of attentional enhancement and suppression across the visual field via amplitude modulation of the early sensory components of the visual ERP.

ERPs evoked by task-relevant stimuli exhibited potentiated N1 amplitude under high perceptual load, relative to low load. This N1 potentiation captured the attentional facilitation of behaviorally relevant visual representations in extrastriate cortex and replicates previous findings of N1 modulation under attentional load [19], [29]-[32]. Attentional facilitation was further indicated by a positive relationship between N1 potentiation and behavioral performance, evidenced by a significant negative correlation between N1 and response time difference scores.

ERPs evoked by irrelevant distractor probes during performance of the attentional load task exhibited modulations of N1 amplitude that varied with distractor eccentricity and interacted with the size of the attended task-relevant stimulus. Three important findings emerged from analyses of distractor probes. First, N1 attenuation was found for irrelevant distractor stimuli within task boundaries whereas N1 enhancement was indicated just outside of task boundaries (see Fig 5). Second, this pattern of suppression and enhancement scaled with the size of the attentional load task, extending more laterally between the 1.0° task and the 2.5° task. Third, distractor suppression within the boundaries of the task-relevant stimulus appears to be most pronounced nearest the inner boundaries of task-relevant space (at least in the 2.5° task). These N1 effects demonstrate clear evidence of the spatial scaling of the distribution of attentional enhancement and suppression of distractor stimuli in the visual field. Taken together, results from task-relevant and distractor ERPs further suggest that, with central attention in a cluttered visual field, attentional selection takes on a more complex pattern than a center-surround configuration.

A number of psychophysical and neuroimaging studies of selective visual attention have reported center-surround organization of enhancement and suppression. In such a configuration, a contiguous facilitatory center region is ringed by a suppressive surround area. Though our ERP results demonstrate clear effects of the spatial scaling of attention, the pattern of results does not conform to a canonical center-surround profile. Quantitative spatial scaling of a center-surround profile of selective attention would predict a more expansive contiguous facilitatory center and broader extending suppressive surround. Rather, attentional effects on the N1 component suggest a more complex configuration in which a pronounced region of suppression overlaps with task-relevant regions of visual space despite a concomitant enhancement of N1 responses from task-relevant stimuli (see Figs 3B and 5A). Suppressing representations of overlapping distractor stimuli is a critical component of visual selection that may serve to enhance the signal-to-noise of an attended stimulus. In a natural visual scene, an attended object is likely to overlap in visual space with assorted distractor stimuli. Such spatially overlapping stimuli would be expected to induce the greatest competition with task-relevant visual representations [3]. Thus, it would be expected that such overlapping stimuli would also experience considerable suppression during selection of task-relevant stimuli. The N1 effects in our study are consistent with such a proposal as the N1 for task-relevant stimuli exhibit enhancement whereas the N1 for overlapping distractor stimuli exhibited suppression. Additionally, the suppression of distractor stimuli within a task-relevant region of space did not appear to be uniform but instead skewed toward the outermost edge of the attended area (Fig 5). This may indicate that (a) distractor suppression occurred upon spatial representations and took on an annulus-shaped distribution [33] or non-contiguous distribution, (b) distractor suppression operated upon spatially invariant object representations, or (c) distractor suppression manifested as an interaction of spatial and object-based selection (e.g., object-guided spatial attention) [34], [35]. The arrangement of distractor stimuli prevent full examination the finer details of spatial attentional configurations. The use annulus stimuli only permit evaluation of changes over eccentricity and do not allow for direct examination of differences between left, right, upper, or lower visual fields. Given overlapping time courses and scalp distributions of N1 during object and spatial selective attention [36], [37] it is impossible to discriminate between these alternatives. However, our results do suggest that distractor suppression is not entirely spatial in nature as there are inverse effects for task-relevant stimuli (enhancement) and distractor probes (suppression) despite their spatial overlap. The operation of selective attention on feature or object-level representations in extrastriate cortex may further point to why attentional effects manifest in N1 but not P1 components [38], [39].

Our results may seem at odds with previous work reporting center-surround profiles for both peripherally and foveally attended stimuli [19], [21], [22], [33], [40]-[43]. However, it is important to note that these previous works have generally constructed spatial profiles from visual probes that were spatially segregated from task-relevant stimuli (however, see [41]) whereas the present investigation used distractor probes that overlapped in visual space with the attended stimulus. This indicates that the spatial pattern of attentional enhancement and suppression is dependent on the visual context in which an attentionally demanding task is performed.

## Conclusion

We sought to examine the spatial scaling of attentional selection across the visual field during a central attentional load task that varied in visual angle (attentional field size). Our results indicate a clear interaction between the visual angle subtended by an attended stimulus and the profile of enhancement and suppression of distractors across the visual field. These findings reveal the occurrence of scaling of attentional selection on representations in extrastriate visual cortex. Our results further suggest that performing a task at fixation in a cluttered visual environment engages a profile of attentional selection more complex than a canonical center-surround configuration that consists of spatially overlapping effects of enhancement and suppression.
